# Bipartite Consensus of Nonlinear Agents in the Presence of Communication Noise

**DOI:** 10.3390/s22062357

**Published:** 2022-03-18

**Authors:** Sabyasachi Mondal, Antonios Tsourdos

**Affiliations:** School of Aerospace, Transport and Manufacturing (SATM), Cranfield University, Cranfield MK43 0AL, UK; e-a.tsourdos@cranfield.ac.uk

**Keywords:** bipartite consensus, Distributed Nonlinear Dynamic Inversion, communication noise, signed graph

## Abstract

In this paper, a Distributed Nonlinear Dynamic Inversion (DNDI)-based consensus protocol is designed to achieve the bipartite consensus of nonlinear agents over a signed graph. DNDI inherits the advantage of nonlinear dynamic inversion theory, and the application to the bipartite problem is a new idea. Moreover, communication noise is considered to make the scenario more realistic. The convergence study provides a solid theoretical base, and a realistic simulation study shows the effectiveness of the proposed protocol.

## 1. Introduction

In the last decade, multiple agents has been considered an attractive area of research for different applications, such as cooperative mobile robotics [1], sensory networks [2], flocking [3], formation control of robot teams [4], rendezvous of multiple spacecraft [5] etc. These agents are connected by a communication network and share information to achieve a common goal cooperatively. The consensus or agreement among agents is the key to successfully attaining the common goal (e.g., the common value of certain dynamic variables). Generally, the consensus is achieved by consensus protocols, which are designed using different branches of control theory.

However, these protocols are designed considering the communication topology represented by a graph. Therefore, the role of the graph is critical. Many researchers have solved different kinds of consensus problems considering communication issues, such as [6,7,8,9,10,11,12,13,14,15,16,17,18,19,20,21] and many more. It is important to note that all these papers show cooperation among the agents, which is analyzed over the nonnegative graph having nonnegative edge weights (antagonistic interactions). However, there should be a way for the agents not to be a part of the consensus and form another group with a different consensus value.

This type of problem was first addressed by Altafini [22] who showed that cooperation and competition are possible over a signed graph with positive and negative edge weights. A single group of agents are divided into two with a consensus value that is the same in magnitude but has an opposite sign. This type of consensus problem is named bipartite consensus. After the bipartite consensus scheme was proposed, there has been an effort to apply the concept to solve different problems in the area, such as a social network and opinion dynamics [23].

Similarly to ordinary consensus, researchers solved various categories of consensus problems for agents with linear dynamics [24,25,26,27,28,29,30,31,32,33,34]. A few researchers experimented with nonlinear agents [35,36,37,38,39,40]. These papers primarily focused on mechanizing a consensus protocol suitable for different types of bipartite consensus problems using different branches of control theory. Along with different control techniques, a nonlinear control technique is popular for designing a nonlinear controller for conventional control problems.

This control technique is known as Nonlinear Dynamic Inversion (NDI) [41]. Recently, a distributed consensus controller was proposed in [21], which was designed using NDI and named Distributed NDI or DNDI. This inherits all the advantages of NDI and is applicable to consensus problems of nonlinear agents. Moreover, DNDI was found to be robust against communication issues, such as noise.

There exist a few papers where the bipartite consensus studied for linear agents considering the noise [42,43,44,45,46,47,48], but none exist (to the best of the authors’ knowledge) for nonlinear agents. DNDI was introduced in the context of ordinary consensus of MASs, and it is not applicable to bipartite problems in its current form. In this paper, we aim to modify the DNDI and make it suitable to apply to bipartite problems of nonlinear agents in the presence of communication noise.

- Feedback linearization theory is used to cancel the nonlinearities in the plant. Moreover, the closed-loop response of the plant is similar to a stable linear system.
- The NDI controller has many advantages. Examples of these advantages include (1) simple and closed-form control expression, (2) easily implementable, global exponential stability of the tracking error, (3) use of nonlinear kinematics in the plant inversion and (4) minimize the need for individual gain tuning, etc.

The contributions of this work are given below:

1. Distributed Nonlinear Dynamic Inversion (DNDI) control protocol is used for bipartite consensus of nonlinear agents for the first time. This is a unique idea because the advantages of NDI are inherited in DNDI and applied to bipartite problems.
2. The mathematical details for the convergence study are presented, which gives a solid theoretical base.
3. The effect of communication noise is studied, which is a practical consideration in the context of multi-agent operation.
4. The detailed simulation study considering the noise separately gives a clear understanding regarding the effectiveness of the proposed consensus protocol.

The rest of the paper is organized as follows. In Section 2, the preliminaries are given. In Section 3, the problem description is presented. Mathematical details of DNDI for bipartite consensus protocol are shown in Section 4. The convergence study of DNDI is presented in Section 5. Simulation results are shown in Section 6, and Section 7 gives our conclusions.

## 2. Preliminaries

A brief description about the topics required for this work is discussed in this section.

### 2.1. Bipartite Consensus of MASs

**Definition** **1.** *A group of agents is said to achieve a bipartite consensus if $\lim_{t\to \infty }\left(x_{i}\left(t\right)-x_{d}\left(t\right)\right)=0,\forall i\in p$ and $\lim_{t\to \infty }\left(x_{j}\left(t\right)+x_{d}\left(t\right)\right)=0,\forall j\in q$, where $x_{d}\left(t\right)$ is a desired trajectory, and p∪q={1,2,…,N};p∩q=∅. It can be mentioned that the definition leads to ordinary consensus when p or q is empty.*

### 2.2. Graph Theory

In this work, we define a weighted graph G={V,E} to represent the communication topology among the agents. The vertices of G are given by $V=\{v_{1},v_{2},\ldots ,v_{N}\}$, which represent the agents. The edges are represented using the set E⊆V×V, which denote the communication among the agents. The connection among the agents are described by an adjacency matrix $A=\left[a_{ij}\right]\in {ℜ}^{N\times N}$. The elements of weighted adjacency matrix A of G are $a_{ij}>0$ if $(v_{i},v_{j})\in E$, otherwise $a_{ij}=0$. Since there is no self loop, the adjacency matrix A has diagonal elements, which are 0, i.e., $v_{i}\in V$, $a_{ii}=0$. The degree matrix is written as $D\in {ℜ}^{N\times N}=diag\left\{d_{1}\;d_{2}\;\ldots d_{N}\right\}$, where $d_{i}=\sum_{j\in N_{i}}a_{ij}$. The Laplacian matrix is written as L=D−A.

The Laplacian matrix L is used to analyze the synchronization of networked agents on a nonnegative graph. However, the Laplacian matrix needs to be defined differently for a signed graph. In the case of a signed graph, $a_{i,j}>0$ means the cooperative interaction, and $a_{i,j}<0$ represents the antagonistic interaction. We define the Laplacian matrix for a signed graph as signed Laplacian ($L_{s}$) given by

(1) $$L_{s}=diag\left(\sum_{j=1}^{N}|a_{1j}|,\ldots ,\sum_{j=1}^{N}\left|a_{1j}\right|\right)-A$$

### 2.3. Communication Noise

The agents share their information over the communication network, but channel noise perturbs them. Therefore the information received by *i*th agent from its neighbours is noisy. In this work, we consider the noise is additive and adopt a noise model, which shows how the noise is added to information shared by the agents with their neighbours. Let us consider the perturbed information received by *i*th agent from *j*th neighbour $j\in N_{i}$ can be given by ${\bar{X}}_{ji}=X_{ji}+\sigma_{ji}\omega_{ji}$, where $X_{i},X_{j}\in {ℜ}^{n}$ are states, $\omega_{ji};\;i,j\in 1,2,\ldots ,N$ are independent standard white noises, and $\sigma_{ji}$ is the noise intensity. This model is used in the simulation study.

### 2.4. Theorems and Lemmas

The useful Lemmas are given here.

**Definition** **2** **((Structural balance) [22,49]).** *A signed graph is structurally balanced if it has a bipartition of the nodes $V_{1}$, $V_{2}$, i.e., $V_{1}\cup V_{2}=V$ and $V_{1}\cap V_{2}=\emptyset$ such that $a_{ij}\leq 0,\forall v_{i}\in V_{p},v_{j}\in V_{q}$ where p,q∈1,2,p≠q, and ∅ is empty set; otherwise $a_{ij}\geq 0$.*

**Lemma** **1** ([50])**.** *A spanning tree is structurally balanced.*

**Lemma** **2** ([51])**.** *Suppose the signed graph G(A) has a spanning tree. Denote the signature matrices set as*

(2) $$D=\{D=diag\left(\sigma_{1},\;\sigma_{2},\;\cdots ,\sigma_{N}\right)|\sigma_{i}\in \left\{1,\;-1\right\}\}$$

*Then the following statements are equivalent.*

1. *G(A) is structurally balanced.*
2. *$a_{ij}a_{ji}\geq 0$ and the associated undirected graph $G(A_{u})$ is structurally balanced, where $G\left(A_{u}\right)=\frac{A+A^{T}}{2}$.*
3. *∃D∈D, such that $\bar{A}=\left[{\bar{a}}_{ij}\right]=DAD$ is a nonnegative matrix.*
4. *either there are no directed semicycles, or all directed semicycles are positive.*

It can be mentioned that the most important property of nonnegative graphs is that when the graph has a spanning tree. In this case, 0 is a simple eigenvalue of the ordinary Laplacian matrix, and all its other eigenvalues have positive real parts ([52]). Some significant results are given for signed digraphs as follows.

**Lemma** **3** ([53])**.** *Suppose the signed digraph G(A) has a spanning tree. If the graph is structurally balanced, then 0 is a simple eigenvalue of its Laplacian matrix and all its other eigenvalues have positive real parts; but not vice versa.*

**Corollary** **1** ([30])**.** *Let G(A) be a nonnegative digraph having a spanning tree. Then for any D∈D, which has both positive and negative entries, the graph G(DAD) is a signed digraph, has a spanning tree and is structurally balanced.*

**Corollary** **2** ([30])**.** *Suppose the signed graph G(A) is undirected and connected. The graph is structurally balanced, if and only if 0 is a simple eigenvalue of L and all other eigenvalues have positive real parts.*

## 3. Problem Description

This paper aims to design a controller to achieve bipartite consensus among the agents in MASs. The communication among the agents is described as a signed digraph G(A), which has a spanning tree and is structurally balanced. The controller is designed by modifying Distributed NDI (DNDI), briefly described in the following section. The dynamics of *i*th agent is given in Equations (3) and (4).

(3) $$\begin{array}{ccc} \dot{X_{i}} & = & f\left(X_{i}\right)+g\left(X_{i}\right)U_{i} \end{array}$$

(4) $$\begin{array}{ccc} Y_{i} & = & X_{i} \end{array}$$

The state and control of *i*th agent is given by $X_{i}\in {ℜ}^{n}$ and $U_{i}\in {ℜ}^{n}$, respectively. The output of *i*th agent is given by

(5) $$Y_{i}=X_{i}\in {ℜ}^{n}$$

The agents are assumed to be working in a randomly changing environment. We considered the communication issues, such as communication noise.

## 4. Distributed Nonlinear Dynamic Inversion (DNDI) Controller for Bipartite Consensus

A derivation of Distributed Nonlinear Dynamic Inversion (DNDI) controller for bipartite consensus is presented in this section. The DNDI is proposed by Mondal et al. [21] for ordinary consensus. In this section, DNDI is modified to achieve bipartite consensus among nonlinear agents. We already mentioned that we consider a signed graph here to analyze the consensus. Therefore, the error in states of *i*th agent (scalar agent dynamics, i.e., $X_{i}\in ℜ$) is given by

(6) $$\epsilon_{i}=\sum_{j\in N_{i}}\left(|a_{ij}|X_{i}-a_{ij}X_{j}\right)$$

Error expression in Equation (6) is simplified to obtain Equation (7).

(7) $$\epsilon_{i}=\left|d_{i}\right|X_{i}-a_{i}X$$

where

$$|d_{i}|=\sum_{j}\left|a_{ij}\right|\in ℜ,\;a_{i}=\left[a_{i1}\;a_{i2}\ldots \;a_{iN}\right]\in {ℜ}^{N}$$

and

$$X=\left[\begin{array}{c} X_{1}\\ X_{2}\\ \vdots \\ X_{N} \end{array}\right]\in {ℜ}^{N}$$

In the case of the state of the *i*th agents being a vector, i.e., $X_{i}\in {ℜ}^{n};n>1$, the error in Equation (7) is modified as

(8) $$\epsilon_{i}=\left|{\bar{d}}_{i}\right|X_{i}-{\bar{a}}_{i}X$$

where $|{\bar{d}}_{i}\left|=(\right|d_{i}\left|⊗I_{n}\right)\in {ℜ}^{n\times n}$, ${\bar{a}}_{i}=\left(a_{i}⊗I_{n}\right)\in {ℜ}^{n\times nN}$, and $X\in {ℜ}^{nN}$. $I_{n}$ is n×n identity matrix. ‘⊗ denotes the kroneker product.

To obtain the consensus protocol, we define a Lyapunov function

(9) $$\Psi =\frac{1}{2}\epsilon_{i}^{T}\epsilon_{i}$$

Differentiation of Equation (9) yields

(10) $$\dot{\Psi }=\epsilon_{i}^{T}{\dot{\epsilon }}_{i}$$

Lyapunov stability condition requires the time derivative of the Lyapunov function to be

(11) $$\dot{\Psi }=-\epsilon_{i}^{T}\kappa_{i}\epsilon_{i}$$

where $\kappa_{i}\in {ℜ}^{n\times n}$ is a positive diagonal gain matrix. Using the expressions of $\dot{\Psi }$ in Equations (10) and (11), we can write

(12) $$\epsilon_{i}^{T}{\dot{\epsilon }}_{i}=-\epsilon_{i}^{T}\kappa_{i}\epsilon_{i}$$

Therefore, Equation (12) is written as

(13) $${\dot{\epsilon }}_{i}+\kappa_{i}\epsilon_{i}=0$$

Expression of $\dot{\epsilon }$ can be obtained by differentiating Equation (8) as follows.

(14) $$\begin{array}{ccc} {\dot{\epsilon }}_{i} & = & |{\bar{d}}_{i}|{\dot{X}}_{i}-{\bar{a}}_{i}\dot{X}\\ & = & |{\bar{d}}_{i}|\left[f\left(X_{i}\right)+g\left(X_{i}\right)U_{i}\right]-{\bar{a}}_{i}\dot{X} \end{array}$$

The expressions of $\epsilon_{i}$ and ${\dot{\epsilon }}_{i}$ are substituted in Equation (13)

(15) $$|{\bar{d}}_{i}|\left[f\left(X_{i}\right)+g\left(X_{i}\right)U_{i}\right]-{\bar{a}}_{i}\dot{X}+\kappa_{i}\left(\right|{\bar{d}}_{i}\left|X_{i}-{\bar{a}}_{i}X\right)=0$$

Finally, Equation (15) is simplified to obtain the expression of $U_{i}$ for *i*th agent as follows

(16) $$U_{i}={\left(g\left(X_{i}\right)\right)}^{-1}\left[-f\left(X_{i}\right)+|{\bar{d}}_{i}{|}^{-1}({\bar{a}}_{i}\dot{X}-\kappa_{i}\left(\right|{\bar{d}}_{i}|X_{i}-{\bar{a}}_{i}X))\right]$$

In the next section, we present the convergence study of the DNDI-based consensus protocol obtained in Equation (16). Before we proceed to the next section, we mentioned a few Lemmas (Lemma 4–6) here, which will be used in the convergence study.

**Lemma** **4** ([54])**.** *The Laplacian matrix L in an undirected graph is semi-positive definite, it has a simple zero eigenvalue, and all the other eigenvalues are positive if and only if the graph is connected. Therefore, L is symmetric and it has N non-negative, real-valued eigenvalues $0=\lambda_{1}\leq \lambda_{2}\leq \ldots \leq \lambda_{N}$.*

**Lemma** **5** ([55])**.** *Let $\psi_{1}\left(t\right),\psi_{2}\left(t\right)\in R^{m}$ be continuous positive vector functions, by Cauchy inequality and Young’s inequality, there exists the following inequality:*

(17) $$\begin{array}{ccc} \psi_{1}\left(t\right)\psi_{2}\left(t\right) & \leq & \|\psi_{1}\left(t\right)\|\|\psi_{2}\left(t\right)\|\\ & \leq & \frac{\|\psi_{1}{\left(t\right)\|}^{\lambda }}{\lambda }+\frac{\|\psi_{2}{\left(t\right)\|}^{\zeta }}{\zeta } \end{array}$$

*where*

$$\frac{1}{\lambda }+\frac{1}{\zeta }=1$$

**Lemma** **6** ([56])**.** *Let R(t)∈ℜ be a continuous positive function with bounded initial R(0). If the inequality holds $\dot{R}\left(t\right)\leq -\beta R\left(t\right)+\eta$ where β>0,η>0, then the following inequality holds.*

(18) $$R\left(t\right)\leq R\left(0\right)e^{-\beta t}+\frac{\eta }{\beta }\left(1-e^{-\beta t}\right)$$

## 5. Convergence Study of DNDI for Bipartite Consensus

Convergence study of DNDI for bipartite consensus is presented here. We define a Lyapunov function

(19) $$\Delta =\frac{1}{2}X^{T}\left(L_{s}⊗I_{n}\right)X$$

We considered a undirected and connected signed graph. Therefore, $L_{s}⊗I_{n}$ can be written as

(20) $$L_{s}⊗I_{n}=\Gamma \Phi \Gamma^{T}$$

where $\Gamma \in {ℜ}^{nN\times nN}$ is the left eigenvalue matrix of $L_{s}⊗I_{n}$, $\Phi =(diag\{0,\lambda_{2}\left(L_{s}\right),\lambda_{3}\left(L_{s}\right),$…,$\lambda_{N}\left(L_{s}\right)\}⊗I_{n})\in {ℜ}^{nN\times nN}$ is eigenvalue matrix, $\Gamma^{T}\Gamma =\Gamma \Gamma^{T}=I_{nN\times nN}$.

(21) $$\begin{array}{ccc} \Delta & = & \frac{1}{2}X^{T}\left(L_{s}⊗I_{n}\right)X\\ & = & \frac{1}{2}X^{T}\Gamma \Phi \Gamma^{T}X\\ & = & \frac{1}{2}X^{T}\Gamma \sqrt{\Phi }\sqrt{\Phi }\;\Gamma^{T}X\\ & = & \frac{1}{2}X^{T}\Gamma \sqrt{\Phi \bar{\Phi }}\sqrt{{\bar{\Phi }}^{-1}}\sqrt{{\bar{\Phi }}^{-1}}\sqrt{\bar{\Phi }\Phi }\;\Gamma^{T}X\\ & = & \frac{1}{2}X^{T}\Gamma \Phi {\bar{\Phi }}^{-1}\Phi \Gamma^{T}X\\ & = & \frac{1}{2}X^{T}\Gamma \Phi \left(\Gamma^{T}\Gamma \right){\bar{\Phi }}^{-1}\left(\Gamma^{T}\Gamma \right)\Phi \Gamma^{T}X\\ & = & \frac{1}{2}X^{T}\left(\Gamma \Phi \Gamma^{T}\right)\left(\Gamma {\bar{\Phi }}^{-1}\Gamma^{T}\right)\left(\Gamma \Phi \Gamma^{T}\right)X\\ & = & \frac{1}{2}X^{T}\left(L_{s}⊗I_{n}\right)\Omega \left(L_{s}⊗I_{n}\right)X\\ & = & \frac{1}{2}\Xi^{T}\Omega \Xi \end{array}$$

where $\bar{\Phi }=\left(diag\left\{\lambda_{2}\left(L_{s}\right),\lambda_{2}\left(L_{s}\right),\lambda_{3}\left(L_{s}\right),\ldots ,\lambda_{N}\left(L_{s}\right)\right\}⊗I_{n}\right)\in {ℜ}^{nN\times nN}$, $\Xi ={\left[\epsilon_{1}^{T}\;\epsilon_{2}^{T}\;\ldots \;\epsilon_{N}^{T}\right]}^{T}\in {ℜ}^{nN}$, and $\Omega =\Gamma {\bar{\Phi }}^{-1}\Gamma^{T}\in {ℜ}^{nN\times nN}$.

**Remark** **1.** *Using Equations (19) and (21), we can write*

(22) $$\frac{\lambda_{min}\left(\Omega \right)}{2}{\left\|\Xi \right\|}^{2}\leq \Delta \leq \frac{\lambda_{max}\left(\Omega \right)}{2}{\left\|\Xi \right\|}^{2}$$

(23) $$\Delta =\frac{1}{2}X^{T}\left(L_{s}⊗I_{n}\right)X=\frac{1}{2}X^{T}\Xi$$

**Remark** **2.** *According to Lemma 4, $\lambda_{2}>0$. Hence, $\bar{\Phi }$ is invertible.*

**Remark** **3.** *It can be observed that $\Omega =\Gamma {\bar{\Phi }}^{-1}\Gamma^{T}$ is positive definite matrix. Therefore, *Δ* is positive definite subject to consensus error and qualify for a Lyapunov function.*

Differentiation of Equation (19) yields

(24) $$\dot{\Delta }=X^{T}\left(L_{s}⊗I_{n}\right)\dot{X}=\Xi^{T}\dot{X}=\sum_{i=1}^{N}\epsilon_{i}^{T}\left[f\left(X_{i}\right)+g\left(X_{i}\right)U_{i}\right]$$

where $\Xi ={\left[\epsilon_{1}^{T}\;\epsilon_{2}^{T}\;\ldots \;\epsilon_{N}^{T}\right]}^{T}\in {ℜ}^{nN}$. Substitution of the control expression of $U_{i}$ in Equation (24) gives

(25) $$\begin{array}{ccc} \dot{\Delta } & = & \sum_{i=1}^{N}\epsilon_{i}^{T}\left[|{\bar{d}}_{i}^{-1}|\left({\bar{a}}_{i}\dot{X}-\kappa_{i}e_{i}\right)\right]\\ & = & \sum_{i=1}^{N}-\epsilon_{i}^{T}|{\bar{d}}_{i}^{-1}|\kappa_{i}\epsilon_{i}+\sum_{i=1}^{N}\epsilon_{i}^{T}\left|{\bar{d}}_{i}^{-1}\right|{\bar{a}}_{i}\dot{X} \end{array}$$

Using Lemma 5, we can write

(26) $$\epsilon_{i}^{T}|{\bar{d}}_{i}^{-1}|{\bar{a}}_{i}\dot{X}\leq \|\epsilon_{i}\left\|\;\|\right|{\bar{d}}_{i}^{-1}\left|{\bar{a}}_{i}\dot{X}\right\|\leq \frac{\|\epsilon_{i}{\|}^{2}}{2}+\frac{\left\|\right|{\bar{d}}_{i}^{-1}{\left|{\bar{a}}_{i}\dot{X}\right\|}^{2}}{2}$$

Substituting $\sum_{i=1}^{N}-\epsilon_{i}^{T}\left|{\bar{d}}_{i}^{-1}\right|\kappa_{i}\epsilon_{i}$ in Equation (25) with inequality relation, we get

(27) $$\dot{\Delta }\leq \sum_{i=1}^{N}\left[-\epsilon_{i}^{T}\left|{\bar{d}}_{i}^{-1}\right|\kappa_{i}\epsilon_{i}+\frac{\|\epsilon_{i}{\|}^{2}}{2}+\frac{\left\|\right|{\bar{d}}_{i}^{-1}{\left|{\bar{a}}_{i}\dot{X}\right\|}^{2}}{2}\right]$$

By designing the gain $\kappa_{i}$ as

(28) $$\kappa_{i}=\left|{\bar{d}}_{i}\right|\left(\frac{1}{2}+\frac{\alpha_{i}}{2}\lambda_{max}\left(\Omega \right)\right)$$

Equation (27) can be written as

(29) $$\begin{array}{ccc} \dot{\Delta } & \leq & \sum_{i=1}^{N}\left[-\frac{\alpha_{i}}{2}\lambda_{max}\left(\Omega \right){\left\|\epsilon_{i}\right\|}^{2}+\frac{\left\|\right|{\bar{d}}_{i}^{-1}|{{\bar{a}}_{i}\dot{X}\|}^{2}}{2}\right]\\ & \leq & -\alpha_{i}\Delta +\zeta \end{array}$$

where $\zeta =\sum_{i=1}^{N}\frac{\left\|\right|{\bar{d}}_{i}^{-1}{\left|{\bar{a}}_{i}\dot{X}\right\|}^{2}}{2}$. Applying Lemma 6 we get

(30) $$\Delta \leq \frac{\zeta }{\alpha_{i}}+\left(\Delta \left(0\right)-\frac{\zeta }{\alpha_{i}}\right)e^{-\alpha_{i}t}$$

Hence, we conclude that Δ is bounded as t→∞. In addition, we show the Uniformly Ultimate Boundedness (UUB) here.

Using Equations ((22)) and ((30)), and Lemma 1.2 presented by Ge et al. in [56] we can write

(31) $$\frac{\lambda_{min}\left(\Omega \right)}{2}{\left\|\Xi \right\|}^{2}\leq \Delta \leq \frac{\zeta }{\alpha_{i}}+\left(\Delta \left(0\right)-\frac{\zeta }{\alpha_{i}}\right)e^{-\alpha_{i}t}$$

Equation (31) is simplified as

(32) $$\begin{array}{ccc} \frac{\lambda_{min}\left(\Omega \right)}{2}{\left\|\Xi \right\|}^{2} & \leq & \frac{\zeta }{\alpha_{i}}+\left(\Delta \left(0\right)-\frac{\zeta }{\alpha_{i}}\right)e^{-\alpha_{i}t}\\ \left\|\Xi \right\| & \leq & \sqrt{\frac{2\frac{\zeta }{\alpha_{i}}+2\left(\Delta \left(0\right)-\frac{\zeta }{\alpha_{i}}\right)e^{-\alpha_{i}t}}{\lambda_{min}\left(\Omega \right)}} \end{array}$$

If $\Delta \left(0\right)=\frac{\zeta }{\alpha_{i}}$, then we can write

(33) $$\left\|\Xi \right\|\leq \Theta^{*}$$

∀t≥0 and $\Theta^{*}=\sqrt{\frac{2\zeta }{\alpha_{i}\lambda_{min}\left(\Omega \right)}}$. If $\Delta \left(0\right)\neq \frac{\zeta }{\alpha_{i}}$ then for any given $\Theta >\Theta^{*}$ there exist a time T>0 such that ∀t>T, ‖Ξ‖≤Θ.

(34) $$\Theta =\sqrt{\frac{2\frac{\zeta }{\alpha_{i}}+2\left(\Delta \left(0\right)-\frac{\zeta }{\alpha_{i}}\right)e^{-\alpha_{i}T}}{\lambda_{min}\left(\Omega \right)}}$$

Therefore, we can conclude

(35) $$\lim_{t\to \infty }\left\|\Xi \right\|=\Theta^{*}$$

## 6. Simulation Study

The simulation results are presented here. We considered two cases. In the first case (Case 1), we describe the performance of DNDI without the communication noise. The second case (Case 2) shows the effect of communication noise.

- Case 1: Bipartite consensus without noise
- Case 2: Bipartite consensus with noise

### 6.1. Agent Dynamics and Control Calculation

We considered six agents in this syudy. The agents are having highly nonlinear terms in their dynamics. The dynamics for *i*th agent [21] is given in Equations (36) and (37).

(36) $$\begin{array}{ccc} {\dot{X}}_{i_{1}} & = & X_{i_{2}}\sin\left(2X_{i_{1}}\right)+U_{i_{1}} \end{array}$$

(37) $$\begin{array}{ccc} {\dot{X}}_{i_{2}} & = & X_{i_{1}}\cos\left(3X_{i_{2}}\right)+U_{i_{2}} \end{array}$$

where $X_{i}={\left[X_{i_{1}}\;X_{i_{2}}\right]}^{T}$. Placing the dynamics of Equations (36) and (37) in the form given in Equations (3) and (4) gives

(38) $$f\left(X_{i}\right)=\left[\begin{array}{c} X_{i_{2}}\sin\left(2X_{i_{1}}\right)\\ X_{i_{1}}\cos\left(3X_{i_{2}}\right) \end{array}\right]$$

and

(39) $$g\left(X_{i}\right)=\left[\begin{array}{cc} 1 & 0\\ 0 & 1 \end{array}\right]$$

and

(40) $$U_{i}=\left[\begin{array}{c} U_{i_{1}}\\ U_{i_{2}} \end{array}\right]$$

where $X_{i}\in {ℜ}^{2}$. The states $X_{1_{i}}$ of all the agents are denoted by $X_{1}=\left[X_{1_{1}}\;X_{2_{1}}\;\ldots \;X_{6_{1}}\right]$. Similarly, we denote $X_{2}=\left[X_{1_{2}}\;X_{2_{2}}\;\ldots \;X_{6_{2}}\right]$, $U_{1}=\left[U_{1_{1}}\;U_{2_{1}}\;\ldots \;U_{6_{1}}\right]$, and $U_{2}=\left[U_{1_{2}}\;U_{2_{2}}\;\ldots \;U_{6_{2}}\right]$. The errors in $X_{1}$ and $X_{2}$ is given by $e_{i}\;in\;X_{1}$ and $e_{i}\;in\;X_{2}$, respectively.

The initial conditions for the agents ($X_{1}$ and $X_{2}$) are given in the Table 1.

### 6.2. Communication Topology

The communication topology is represented by a signed graph. The adjacency matrix corresponding to the graph is given in Equation (41).

(41) $$A=\left[\begin{array}{cccccc} 0 & 3 & 0 & -5 & 0 & 1\\ 3 & 0 & -4 & 0 & 0 & 1\\ 0 & -4 & 0 & 0.5 & 0 & 0\\ -5 & 0 & 0.5 & 0 & -3.5 & 0\\ 0 & 0 & 0 & -3.5 & 0 & 1\\ 1 & 1 & 0 & 0 & 1 & 0 \end{array}\right]$$

**Figure 1:**
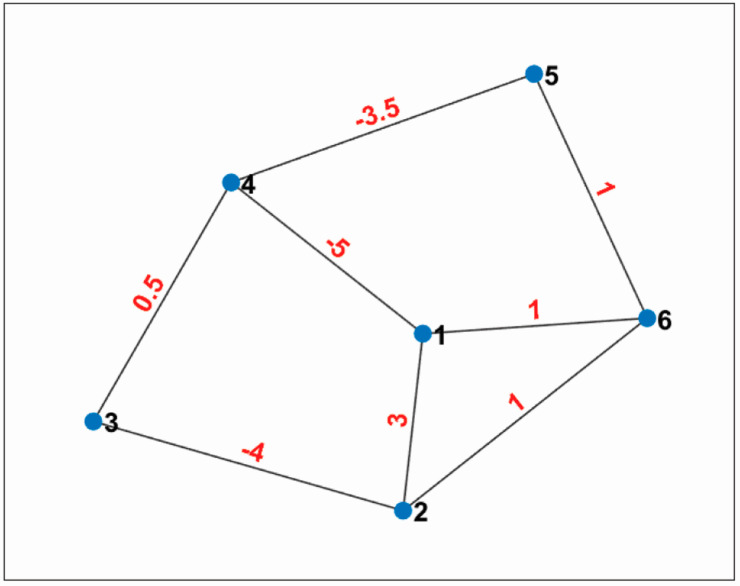
Signed graph corresponding to A.

**Figure 2:**
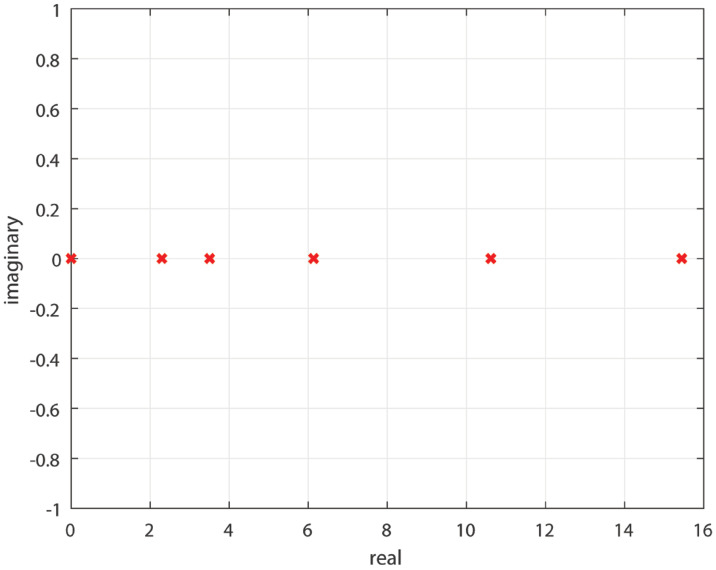
Eigen values of signed graph.

The graph corresponding to the adjacency matrix is shown in Figure 1. The weights are on each edge. The signed graph is undirected and connected. The eigenvalues of the Laplacian matrix ($L_{s}$) of this signed graph are shown in Figure 2. One eigenvalue is zero and the other have a positive real part. Therefore, the graph has a spanning tree, and it is structurally balanced (Corollary 2).

### 6.3. Case 1: Bipartite Consensus without Noise

**Figure 3:**
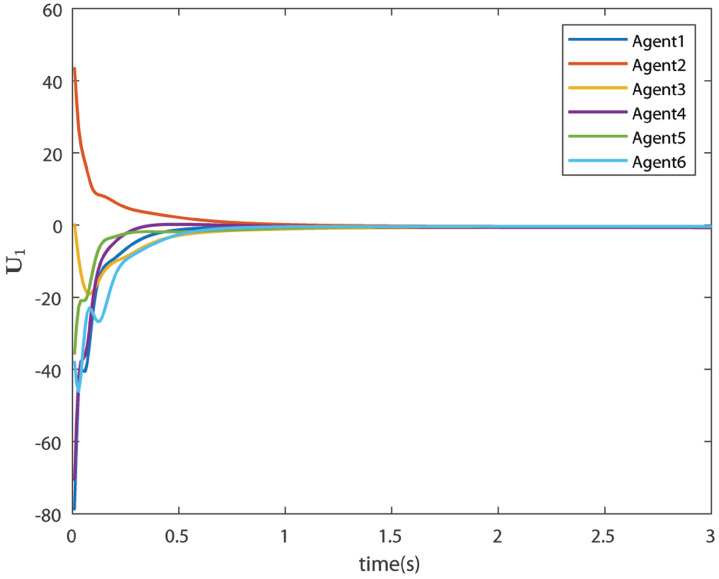
Control $U_{1}$ (Case 1).

**Figure 4:**
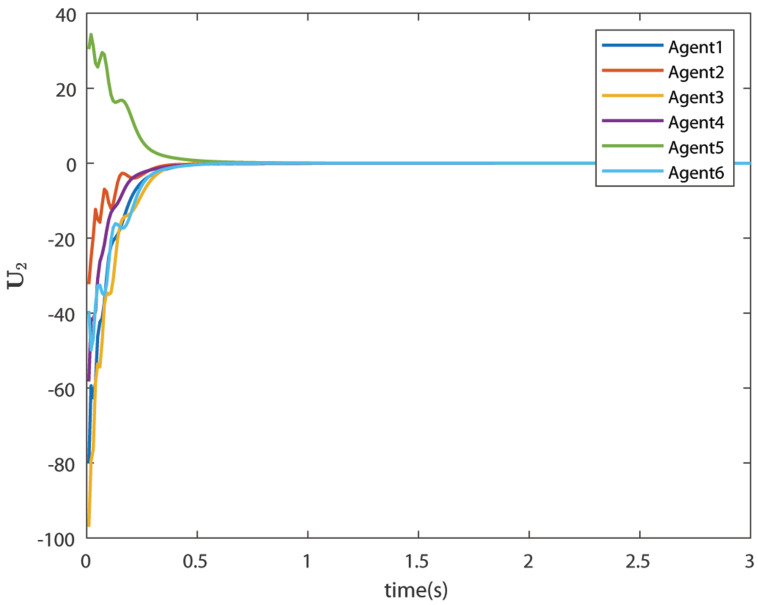
Control $U_{2}$ (Case 1).

**Figure 5:**
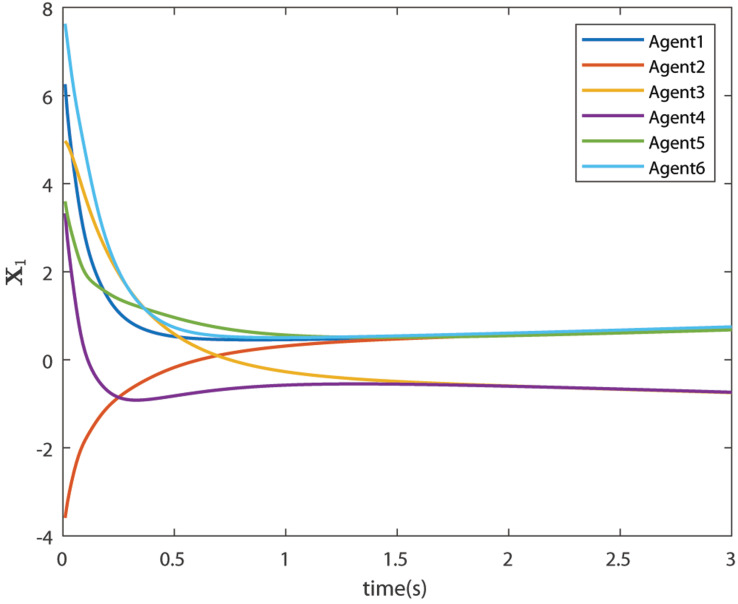
States of the agents $X_{1}$ (Case 1).

**Figure 6:**
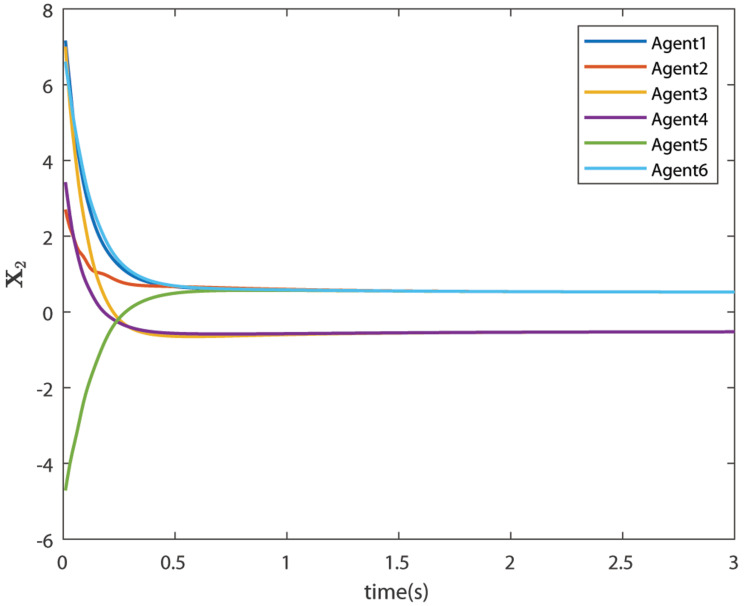
States of the agents $X_{2}$ (Case 1).

**Figure 7:**
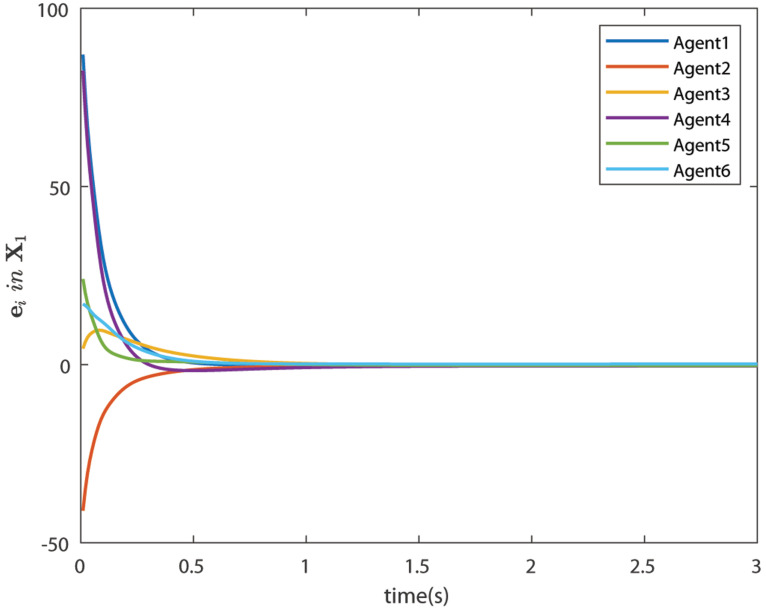
Consensus errors of agents in state $X_{1}$ (Case 1).

**Figure 8:**
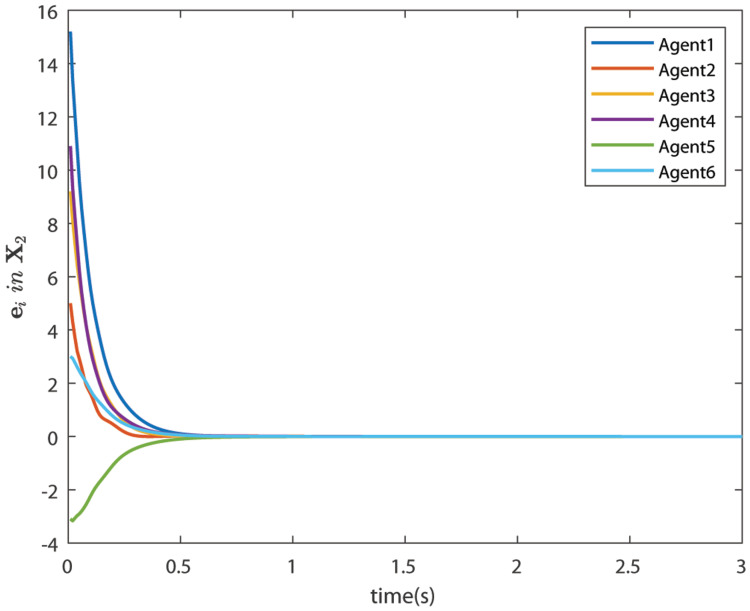
Consensus errors of agents in state $X_{2}$ (Case 1).

The control signals $U_{1}$ and $U_{2}$ obtained by DNDI are given in Figure 3 and Figure 4, respectively. These controls have generated the bipartite consensus among the agents. It can be observed that the states of the agents are divided into two groups. This is primarily for the signed graph and the consensus protocol used in this work. One group contains the agents 1, 2, 5, and 6. The other group contains agents 3 and 4. The states of all the agents, i.e., $X_{1}$ and $X_{2}$ are shown in Figure 5 and Figure 6, respectively. It is clear that the states of agents in each group achieved the consensus with different values. The consensus errors in states $X_{1}$ and $X_{2}$ are shown in Figure 7 and Figure 8, respectively. The errors converge to zero in a few seconds, which shows the effectiveness of the proposed controller.

### 6.4. Case 2: Bipartite Consensus with Noise

**Figure 9:**
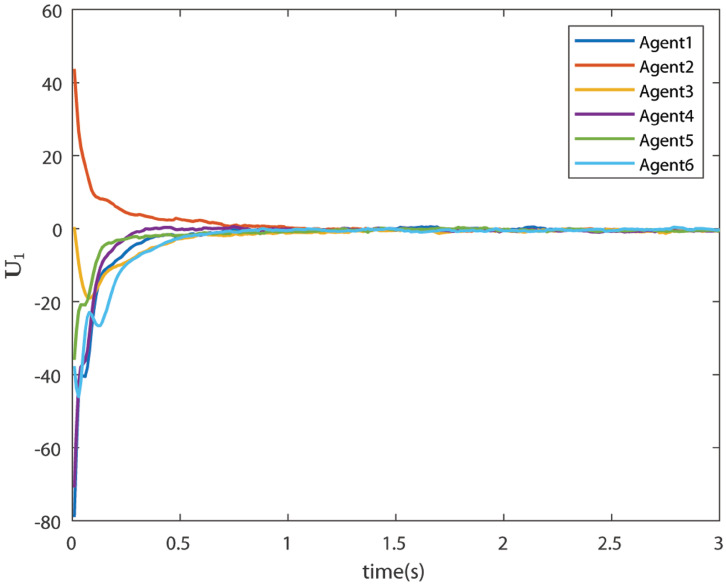
Control $U_{1}$ (Case 2).

**Figure 10:**
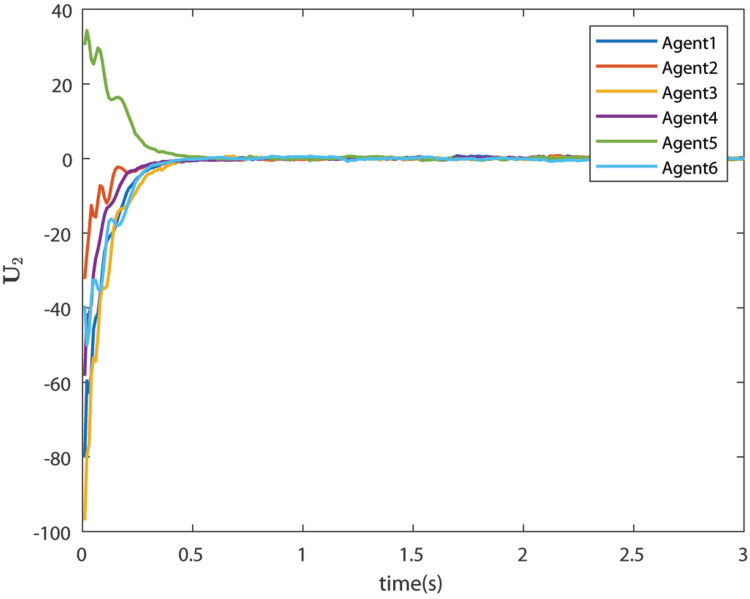
Control $U_{2}$ (Case 2).

**Figure 11:**
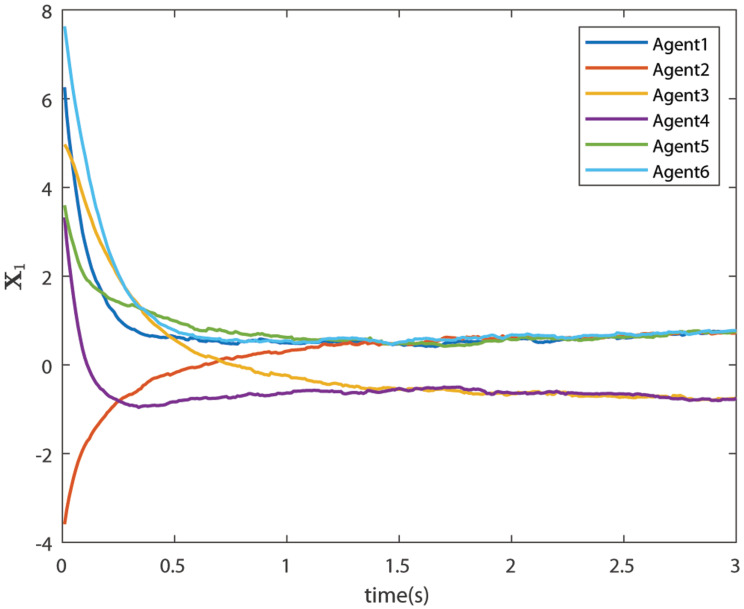
States of the agents $X_{1}$ (Case 2).

**Figure 12:**
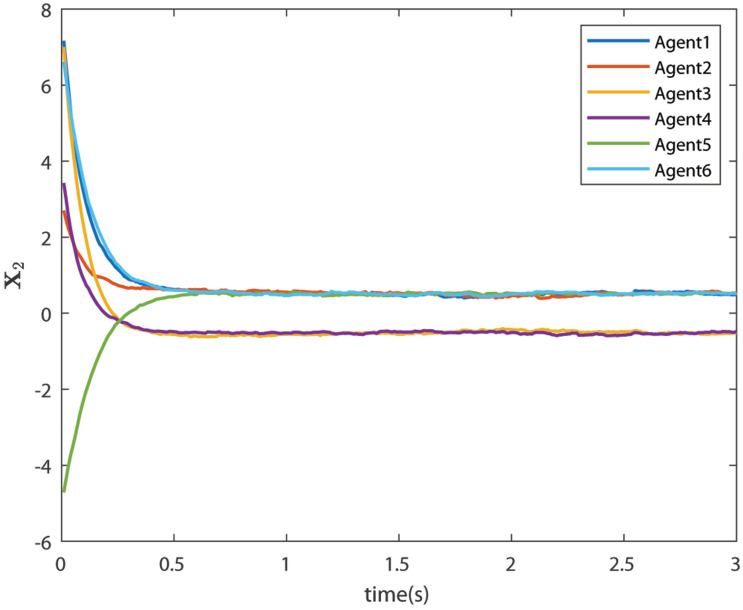
States of the agents $X_{2}$ (Case 2).

**Figure 13:**
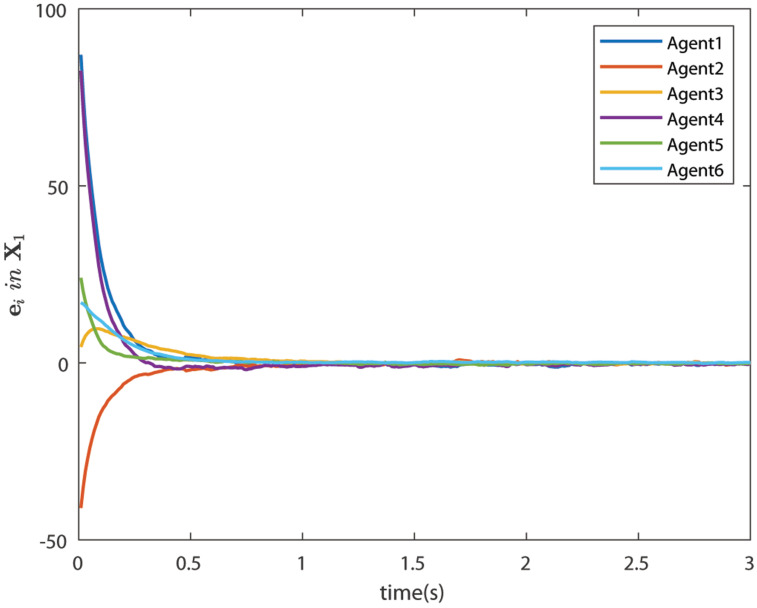
Consensus errors of agents in state $X_{1}$ (Case 2).

**Figure 14:**
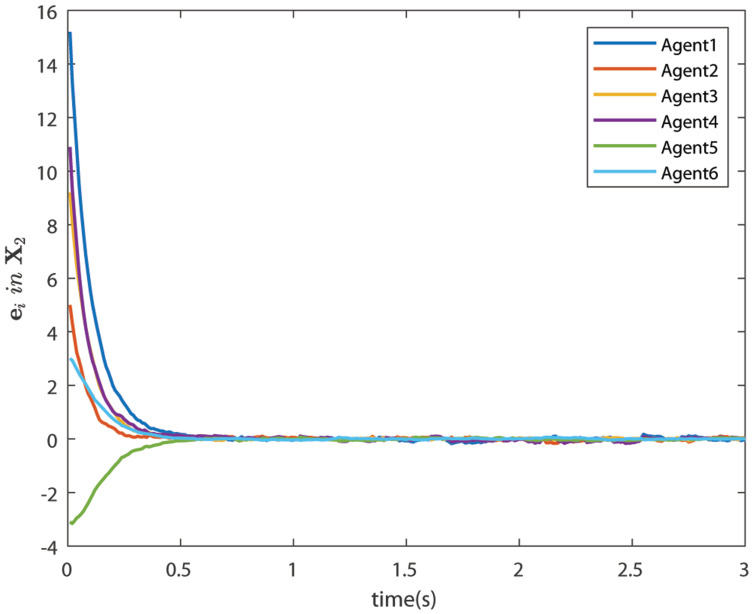
Consensus errors of agents in state $X_{2}$ (Case 2).

In this case, the effect of communication noise is studied. The control signals $U_{1}$ and $U_{2}$ are given in Figure 9 and Figure 10, respectively. The figures show the effect of communication noise. The noise intensity is considered as $\sigma_{ji}=0.25\times rand\left(\right)$, where rand() is a MATLAB function, which generates random number between 0 and 1. The effect of communication noise on states $X_{1}$ and $X_{2}$ is shown in Figure 11 and Figure 12, respectively. The consensus errors (Figure 13 and Figure 14) confirms the performance of the DNDI controller. Therefore, it is clear that the proposed controller is able to achieve the bipartite consensus in the presence of communication noise.

## 7. Conclusions

We modified the DNDI controller to achieve bipartite consensus among nonlinear agents. The application of DNDI in the bipartite consensus problem is a new idea. We also included communication noise in the simulation study, which is realistic. The convergence study showed the theoretical proof of the effectiveness of the controller. The simulation results provided in the paper show the assured performance of the proposed controller. Therefore, DNDI is a potential candidate for achieving bipartite consensus among nonlinear agents. 

## Figures and Tables

**Table 1:** The initial conditions of the agents.

Agents	1	2	3	4	5	6
$X_{10}$	6.25	−3.6	5	3.3	3.6	7.6
$X_{20}$	7.2	2.7	7	3.4	−4.7	6.6

## Data Availability

No new data were created or analyzed in this study. Data sharing is not applicable to this article.

## Abbreviations

The following abbreviations are used in this manuscript:

DNDI	Distributed Nonlinear Dynamic Inversion
N-DNDI	Neuro-adaptive augmented DNDI
